# Mechanistic Insights into the Membrane Permeabilization Activity of Antimicrobial Prenylated Isoflavonoids: A Comparative Study of Glabridin, Wighteone, and Lupiwighteone

**DOI:** 10.1021/acs.jafc.5c01688

**Published:** 2025-03-05

**Authors:** Alberto Bombelli, Paolo Calligari, Gianfranco Bocchinfuso, Jean-Paul Vincken, Tjakko Abee, Heidy M.W. den Besten, Lorenzo Stella, Carla Araya-Cloutier

**Affiliations:** † Food Microbiology, Wageningen University & Research, AA Wageningen 6700, the Netherlands; ‡ Food Chemistry, Wageningen University & Research, AA Wageningen 6700, the Netherlands; § Department of Chemical Science and Technologies, Tor Vergata University of Rome, Rome 00133, Italy

**Keywords:** mode of action, simulation, molecular dynamics, liposomes, phospholipid

## Abstract

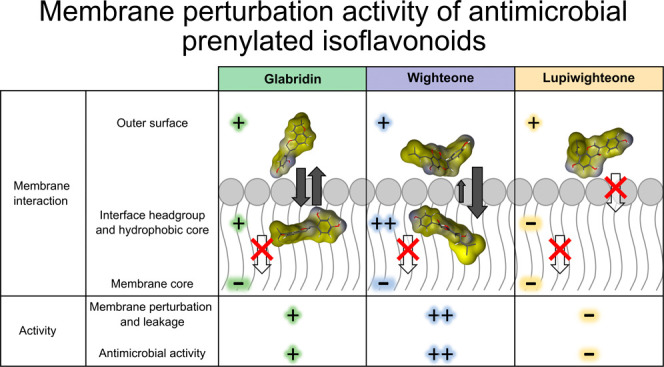

Prenylated isoflavonoids have shown remarkable antimicrobial activity. Previous studies showed that the antimicrobial compounds glabridin and wighteone induced membrane permeabilization in microbial cells. Other compounds, such as lupiwighteone, showed no antimicrobial activity. In this study, the permeabilization efficacy and interaction with lipid bilayers of glabridin, wighteone, and lupiwighteone were assessed *in vitro* and *in silico* using model membranes. Permeabilization of liposomes by glabridin and wighteone confirmed the lipid bilayer as the primary target. Notably, lupiwighteone did not induce the permeabilization of liposomes. Molecular dynamics (MD) simulations were used to study the interaction of these compounds with phospholipid membranes. The calculated potential of mean force profiles for the three molecules correlated with liposome permeabilization, indicating a favorable intercalation inside the lipid bilayer for wighteone, followed by glabridin, and an unfavorable intercalation for lupiwighteone. Additionally, MD simulations indicated that the location of glabridin and wighteone in the membrane was just below the head groups. Furthermore, this study underscored the importance of partitioning between polar and hydrophobic areas for prenylated isoflavonoids, which conceivably determines the membrane insertion and, subsequently, the antimicrobial activity. Overall, this study showed that interactions with and permeabilization of the lipid bilayer are key factors for the antimicrobial activity of these compounds.

## 1 Introduction

The need and interest in new natural antimicrobial compounds in various sectors, such as food and pharma, have driven researchers to explore plants as natural sources of antimicrobial agents.^1^ Flavonoids and isoflavonoids are phytochemicals known to exhibit interesting bioactivities, including antimicrobial activity,^2^ and can be modified with different functional groups. One interesting example of this modification is called prenylation, the addition of a 5-carbon isoprenoid group called prenyl. Prenylated isoflavonoids can be present in plants of the Fabaceae family, and it has been shown that abiotic and biotic stresses induce an increased production of prenylated isoflavonoids.^3−5^ This prenyl addition is a crucial diversification and bioactivation step for this class of compounds. Prenylated isoflavonoids show increased biological activity compared to their unsubstituted counterparts, including higher antimicrobial activity.^6^ For example, in a screening of 85 flavonoids for their antimicrobial activity against *Staphylococcus aureus*, all 37 active compounds were prenylated.^7^ Moreover, previous antimicrobial screening of a large number of prenylated isoflavonoids has led to the identification of highly active compounds such as glabridin and wighteone.^8−10^

**Figure 1:**
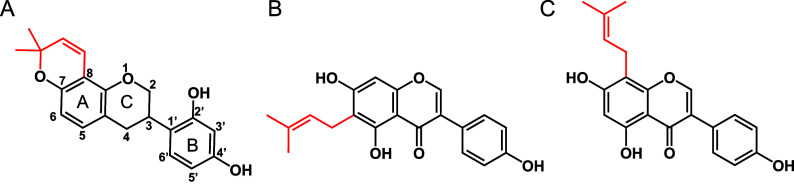
Prenylated isoflavonoids tested in this study. Chemical structure of glabridin (**A**), wighteone (**B**), and lupiwighteone (**C**). The prenyl group is highlighted in red. The subclass-dependent IUPAC numbering shown on glabridin is also applicable for the other compounds.

Glabridin is a prenylated isoflavan with a prenyl group at position *C*8, which forms a six-membered ring by coupling to a neighboring hydroxyl group (Figure 1A). Glabridin can be extracted from licorice (*Glycyrrhiza glabra*) roots^11^ and has potent antimicrobial activity against various microorganisms, including Gram-positive bacteria, such as *Listeria monocytogenes*, *Bacillus subtilis*, and methicillin-resistant *Staphylococcus aureus* (MRSA), and yeast cells such as *Zygosaccharomyces parabailii*.^8−10,12^ The minimum inhibitory concentrations (MIC) against these microorganisms range from 19 μM to 39 μM, making this natural compound of great interest. Moreover, we previously showed that glabridin has potential application as an antimicrobial in the food industry, for example, as a food preservative or disinfectant against *L. monocytogenes*.^13,14^ Wighteone is another antimicrobial prenylated isoflavonoid (isoflavone), which can be extracted from blue lupine (*Lupinus angustifolius*);^15^ it is derived from genistein by the addition of the prenyl group in position *C*6 (chain prenylation, Figure 1B). It has been shown that wighteone is also a potent antimicrobial against Gram-positive and yeast cells, with MIC values ranging from 9 to 30 μM.^8,10^ Glabridin and wighteone, like other prenylated (iso)flavonoids, showed limited activity against Gram-negative bacteria (e.g., *Escherichia coli*), except when combined with an efflux pump inhibitor, indicating that efflux is a key resistance mechanism in Gram-negative bacteria.^8^

The antimicrobial activity of prenylated isoflavonoids is highly structure dependent; i.e., it can be affected by the presence and/or position of different substitutions, such as hydroxyl, methyl, and prenyl groups. An interesting example is the difference in activity between wighteone (Figure 1B) and lupiwighteone (Figure 1C). The only structural difference between these compounds is the position of the prenyl group; lupiwighteone is prenylated at position *C*8 instead of *C*6. This subtle change in the chemical structure induces drastic changes in the antimicrobial properties as lupiwighteone is inactive against Gram-positive and Gram-negative bacteria and yeast cells, making this a general effect rather than target specific.^8,9,16^ Although studies have reported this inactivity, a mechanistic explanation for this drastic reduction in activity is yet to be provided.

The antimicrobial activity of prenylated isoflavonoids has been related to possible interaction with microbial cytoplasmic membranes and subsequent permeabilization. For example, we have previously indicated the cell membrane as the primary target of glabridin as an antimicrobial against *L. monocytogenes* using fluorescence and electron microscopy.^17^ Moreover, membrane permeabilization has also been tested for glabridin and wighteone against various microorganisms such as *L. monocytogenes*, *E. coli*, and *Z. parabailii*.^8,9^ No studies have tested the membrane permeabilization of microbial cells upon exposure to lupiwighteone. Due to the molecular characteristics of prenylated isoflavonoids and previous research on similar compounds, the lipid part of the membrane has been identified as a possible target of the prenylated isoflavonoids glabridin and wighteone.^8,18^ However, further research is necessary to verify and comprehend this suggested interaction.

Bacterial membranes are complex structures with approximately equivalent proportions of lipids and proteins.^19^ Whole-cell assays, such as propidium iodide uptake and fluorescence microscopy, are pivotal steps in investigating the mode of action of membrane-active antimicrobial compounds. However, using these assays to characterize the activity on the lipid part of the membranes has some limitations; membrane permeabilization may also occur after interactions with membrane proteins or as a secondary effect due to intracellular mechanisms. Notably, *in vitro* and *in silico* model membrane approaches rely on methodologies in which membranes are represented as simplified systems containing only the lipid bilayers.^20,21^ These techniques can be used to confirm the activity of a compound at the lipid level due to the absence of intracellular microbial metabolism and other components in the membrane (such as proteins).

Liposomes are spherical lipid vesicles that can be used *in vitro* to simulate the lipid bilayer of membranes.^20^ Liposomes are also suitable for investigating the membrane permeabilization activity of tested compounds by entrapping a fluorescent dye inside the liposome and measuring an increase in the fluorescent signal due to the release of the dye.^22 ,23^ Combining experimental data and *in silico* methods has often provided significant insights into the molecular mechanisms underlying biological activity.^24−26^ In this context, molecular dynamics (MD) simulations have been widely used to study small molecule-membrane interactions and have been shown to be suitable for predicting affinities and molecular interactions on an atomic scale.^27^ In particular, the calculation of the potential of mean force (PMF) profile of compounds based on specific positions in the membrane has been shown to simulate the intercalation of compounds in the membrane, identify energy barriers, and estimate favorable positions and conformations.^27^ Model membrane approaches have been previously used to study the interaction of prenylated flavonoids, such as 6,8-diprenylgenistein and mangostin,^28−30^ and antimicrobial peptides.^31,32^ Although the importance of the prenyl position has already been reported as a key feature for antimicrobial activity, differences in membrane interaction and intercalation based on the prenyl position of isoflavonoids have not yet been investigated.

In this study, we assessed the permeabilization activity of glabridin, wighteone, and lupiwighteone with liposomes prepared from *E. coli* and *S. cerevisiae* lipid extracts and selected synthetic phospholipids. Moreover, *in silico* MD simulation studies were implemented to investigate the interactions of the selected prenylated isoflavonoids with the phospholipid bilayer. Due to the subtle difference between wighteone and lupiwighteone underlying the change in bioactivity, lupiwighteone was included to investigate if this inactivity is also present in model membranes and to understand the impact of different prenyl positions on the interaction with membranes. Overall, this study aims to understand the interaction of prenylated isoflavonoids with membranes to further explain their structure–function relations and mode of action as antimicrobial compounds.

## 2 Materials and Methods

### 2.1 Broth Microdilution Assay

Antibacterial activity was determined using the broth microdilution assay at 37 °C, as described by Bombelli et al. (2023)^13^ for *L. monocytogenes* EGDe and at 30 °C as described by Kalli et al. (2022)^9^ for *S. cerevisiae* S288C. The minimum inhibitory concentration (MIC) and the minimum bactericidal concentration (MBC) were defined based on cell counts. The MIC is the lowest concentration that inhibits growth (i.e., no increase in cell concentration after 24 h), and the MBC is the lowest concentration at which at least 3 log_10_ reductions were obtained.

### 2.2 Unilamellar Liposome Preparation

Liposomes were prepared with two lipid extracts, *E. coli* and *S. cerevisiae* total lipid extract, and a mixture of POPC (1-palmitoyl-2-oleoyl-glycero-3-phosphocholine) and POPG (1-palmitoyl-2-oleoyl-*sn*-glycero-3-(phospho-rac-(1-glycerol))) at a 2:1 molar ratio. The total lipid extracts and the pure lipids were purchased from Avanti Polar Lipids (Alabaster, AL, USA). The composition of *E. coli* and *S. cerevisiae* total lipid extract is reported in Table S1. Lipids were resuspended in chloroform and subsequently dried with a rotavapor and then by freeze-drying overnight. The lipid film was hydrated with 10 mM phosphate buffer (pH 7.4) containing 30 mM 5(6)-carboxyfluorescein (Sigma-Aldrich, St. Louis, MO, USA) and 80 mM sodium chloride to make it isotonic to the dilution buffer (according to Bortolotti et al. (2023).^21^ The dilution buffer used was 10 mM phosphate buffer with 140 mM sodium chloride and 0.1 mM EDTA (pH 7.4). The hydrated lipids were extruded 21 times through a polycarbonate membrane with 100 nm pores (Avanti Polar Lipids), and the unencapsulated dye was separated by gel filtration (Sephadex G-50, Marlborough, MA, USA). Hydration and extrusion were performed at 35 °C. The lipid concentration was quantified with the Stewart assay^33^ and adjusted to reach a final test concentration in the carboxyfluorescein leakage assay of 50 μM for POPC:POPG and 35 μg/mL for the lipid extracts, which is equal to 50 μM of lipids, assuming 700 g/mol as an average lipid molecular weight.^34^ The correct size of the liposomes was confirmed by dynamic light scattering (DLS) measurements (Figure S1).

### 2.3 Carboxyfluorescein Leakage

To assess the membrane leakage upon exposure to prenylated isoflavonoids, the release of carboxyfluorescein from the liposomes was measured by an increase in fluorescence intensity. Carboxyfluorescein is present in the liposomes at a self-quenching concentration (30 mM);^35^ therefore, an increase in fluorescence indicates the release of carboxyfluorescein from the liposomes and its dilution into the external solution.^23^ Glabridin and lupiwighteone (reported purity ≥99%) were purchased from ChemFaces (Wuhan, China), and wighteone (reported purity ≥99%) was purchased from MedChemTronica (Sollentuna, Sweden). Stock solutions (10 mg/mL) of prenylated isoflavonoids were prepared in DMSO. Liposomes and prenylated isoflavonoids were diluted in 10 mM phosphate buffer with 140 mM sodium chloride and 0.1 mM EDTA (pH 7.4). Equal volumes (100 μL) of liposomes and prenylated isoflavonoids were mixed in a black plate with a clear bottom. Fluorescence was recorded every minute with a Spectramax ID3 (Molecular Devices, San Jose, CA, USA) for 30 min at 25 °C. Negative controls (addition of 100 μL of phosphate buffer) and positive controls (liposomes treated with 1% Triton X-100) were included in every experiment. The fractional release was calculated with eq 1:
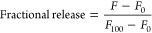
1where F is the fluorescent measurement of the sample; F_0_ and F_100_ are the averages of the last 5 min of incubation for the negative and positive controls, respectively. DMSO 1% (maximum concentration tested) did not induce permeabilization of the liposomes (Figure S2), confirming the suitability of this solvent to investigate the permeabilization activity of prenylated isoflavonoids in liposomes.

### 2.4 MD Simulations

#### 2.4.1 Preparation of the System

Topology and structure files for glabridin, wighteone, and lupiwighteone (united atoms) were generated using ATB,^36^ which ensures compatibility with the GROMOS 54A7 force field. For the MD simulations, the neutral form of prenylated isoflavonoids was used. The hydrophobic chains in POPC and POPG were modeled with the Berger parameters.^37^ MemGen^38^ was used to build the initial bilayer structure containing 128 phospholipid molecules (86 POPC and 42 POPG). Further preparation of the system and simulations were performed using the GROMACS 2021 software package and the GROMOS 54A7 force field. The obtained membrane bilayer was placed at the center of a new box, with dimensions tailored for subsequent umbrella sampling simulations and solvated with SPC/E water molecules. Na^+^ counterions were added to neutralize the total net charge. The overall system was then energetically minimized and equilibrated to 25 °C and 1 atm. Last, 500 ns of simulation were produced to obtain the system used to study the interaction of prenylated isoflavonoids with the membrane. Details of the MD simulation systems used in this study are presented in Tables S2 andS3.

#### 2.4.2 Potential of Mean Force (PMF)

The potential of mean force (PMF) free energy profiles were calculated using umbrella-sampling simulations. The centers of mass (c.o.m) of glabridin, wighteone, or lupiwighteone were positioned at 5 nm from the center of mass of the bilayer (*z*-axis) (Figure S3). Prenylated isoflavonoids were pulled from the starting position (z = 5 nm) to slightly beyond the center of the membrane (z = −0.5 nm) with a pulling rate of 0.1 Å/ns and a force constant of 3,000 kJ/mol nm^2^ (adapted from Daison et al. (2022)).^39^ In total, 102 windows were generated from the trajectory from z = 4.55 to z = −0.5 nm, with a step size of 0.05 nm distance. Each window was subjected to 40 ns of simulation, including an annealing step (as reported by Farrotti et al. (2017),^40^Table S3), with a restraining force constant of 3,000 kJ/mol nm^2^. The last 15 ns of simulation of each window were used to calculate the PMF profile using the Weighted Histogram Analysis Method (WHAM).^41^ The annealing step was conducted to equilibrate the system; the validation of this equilibration was made by comparing the PMF obtained with the simulation produced before and after the annealing step and the profile obtained in the production step. The convergence to a similar PMF profile indicated the correct equilibration of the system (as further described in Figure S4).

#### 2.4.3 Clustering

The clustering of selected structures was used to identify the most representative configuration of the prenylated isoflavonoids tested in the bilayer. The simulation frames were selected based on the distance from the center of the membrane; specifically, frames where the c.o.m. of the prenylated isoflavonoids were at a distance of 2.75 ± 0.05 and 1.45 ± 0.05 nm were retrieved. These two distances represent situations where the compounds are present on the surface of the membrane and when they are intercalated below the headgroup of the membrane, respectively. Moreover, these specific ranges correspond to the minima in the PMF profile of wighteone, as shown in the Results section. The most representative structure of prenylated isoflavonoids was identified by applying the single linkage clustering method with a 0.5 nm cutoff on the selected conformations after removal of the translational motion along the xy plane.

#### 2.4.4 Visualization

Visualization of the simulations was done using Chimera 1.17.3.^42^ Molecular Operating Environment (MOE) software (version 2019.0102, Chemical Computing Group) was used to visualize the molecular surface of prenylated isoflavonoids and to calculate the molecular descriptors.

## 3 Results

### 3.1 Antimicrobial Activity of Glabridin, Wighteone, and Lupiwighteone

The antimicrobial activity of glabridin, wighteone, and lupiwighteone was assessed with the broth microdilution assay against *L. monocytogenes* and *S. cerevisiae* (Table 1). For comparison, activity against *E. coli* in the presence of an efflux pump inhibitor (EPI) is also reported in the table (values according to Araya-Cloutier et al. (2018).^8^ Glabridin and wighteone showed potent antimicrobial activity against the microorganisms tested, with MIC values ranging from 18 to 77 μM (6.25–25 μg/mL) and MBC values ranging from 37 to 154 μM (12.5–50 μg/mL). Notably, lupiwighteone was inactive as an antimicrobial agent at the maximum concentration tested, 296 μM (100 μg/mL).

**Table 1:** Minimum Inhibitory Concentrations (MIC) and Bactericidal Concentrations (MBC, between Brackets) of Glabridin, Wighteone, and Lupiwighteone

	**MIC (MBC) μM**^a^		
	Glabridin	Wighteone	Lupiwighteone
*L. monocytogenes*	39 (54)	18 (37)	>296
*S. cerevisiae*	77 (154)	37 (74)	>296
*E. coli* (+EPI)^b^	31 (46)	44 (44)	>148

a Compounds were tested with 2-fold dilution concentrations from 3.1 to 100 μg/mL; MIC and MBC values converted to corresponding μM concentrations (molecular weight of 324.4 g/mol for glabridin and 338.4 g/mol for wighteone and lupiwighteone).

b The activity of prenylated isoflavonoids against *E. coli* was according to Araya-Cloutier et al. (2018)^8^ where compounds were tested in the presence of the efflux pump inhibitor PaβN (EPI, 48 μM).

### 3.2 Liposome Permeabilization Efficacy

Membrane permeabilization by glabridin, wighteone, and lupiwighteone was investigated by assessing the release of carboxyfluorescein from unilamellar liposomes.

**Figure 2:**
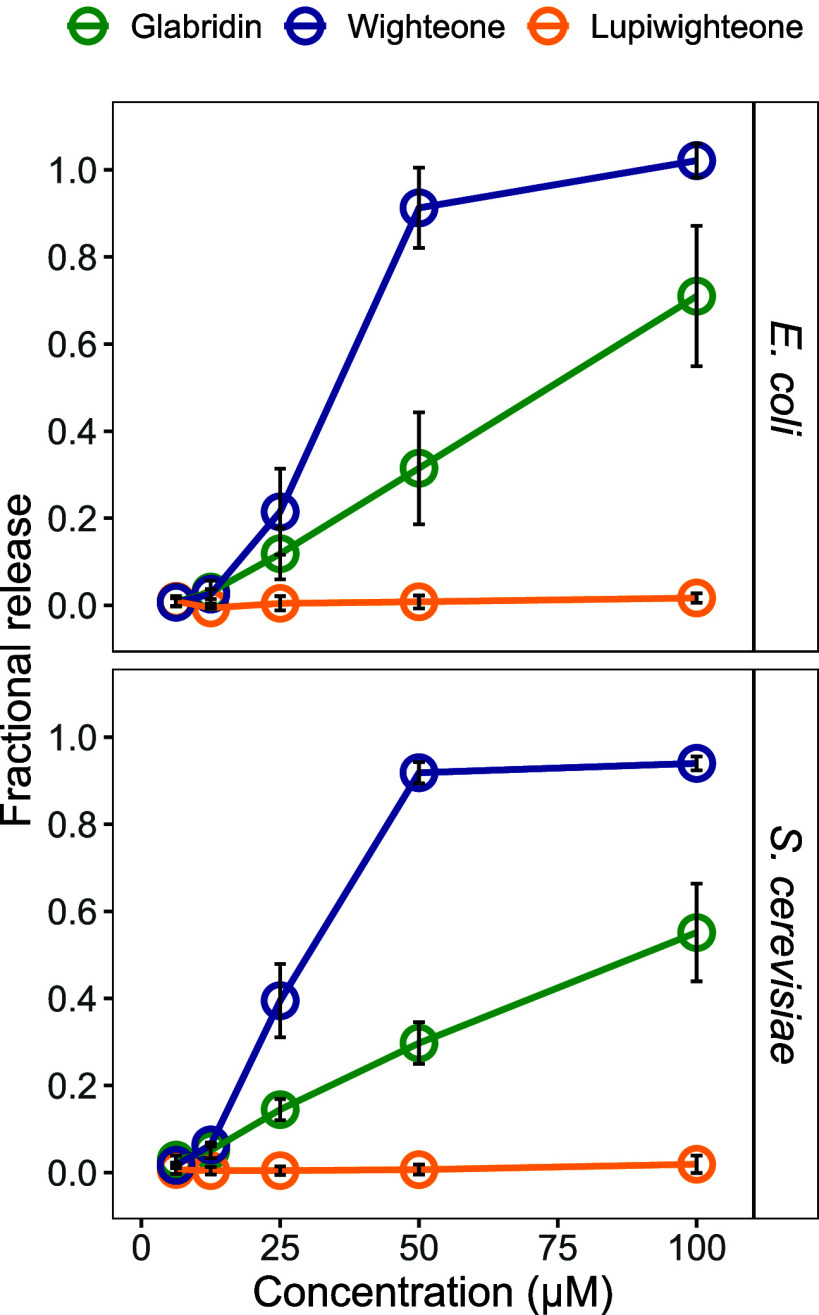
Fractional release of carboxyfluorescein from liposomes formed with *E. coli* (**top**) and *S. cerevisiae* (**bottom**) lipid extract after 30 min of incubation with glabridin (green), wighteone (blue), and lupiwighteone (yellow). Data are expressed as averages, and error bars represent the standard deviation of independent replicates (*n* = 3).

First, liposomes were prepared with *E. coli* and *S. cerevisiae* total lipid extract to closely simulate the assays used to test their antimicrobial activity against bacterial and yeast cells. Figure 2 shows the fractional release of carboxyfluorescein after 30 min of incubation with different concentrations of prenylated isoflavonoids. Wighteone showed the highest permeabilization, both with the *E. coli* and *S. cerevisiae* extracts. The release of carboxyfluorescein occurred with concentrations of wighteone above 12.5 μM. Specifically, 25 μM of wighteone induced 0.2 ± 0.1 and 0.4 ± 0.1 fractional release when assessed with *E. coli* and *S. cerevisiae* liposomes, respectively. Maximum leakage occurred after 30 min of exposure in the presence of 50 μM of wighteone. The release curves (Figure S5) further demonstrate the fast permeabilization effect of wighteone on liposomes.

Glabridin also induced permeabilization with concentrations above 12.5 μM in liposomes prepared with *E. coli* and *S. cerevisiae* lipid extracts (Figure 2). At the highest tested concentration (100 μM), glabridin induced a fractional release of 0.7 ± 0.2 and 0.6 ± 0.1 in *E. coli* and *S. cerevisiae* liposomes, respectively. In comparison to wighteone, glabridin was shown to induce less permeabilization in *E. coli* and *S. cerevisiae* liposomes (Figure 2) with overall slower activity (Figure S5). Notably, lupiwighteone did not induce permeabilization of *E. coli* and *S. cerevisiae* liposomes even at the highest concentration tested (100 μM) (Figure 2).

**Figure 3:**
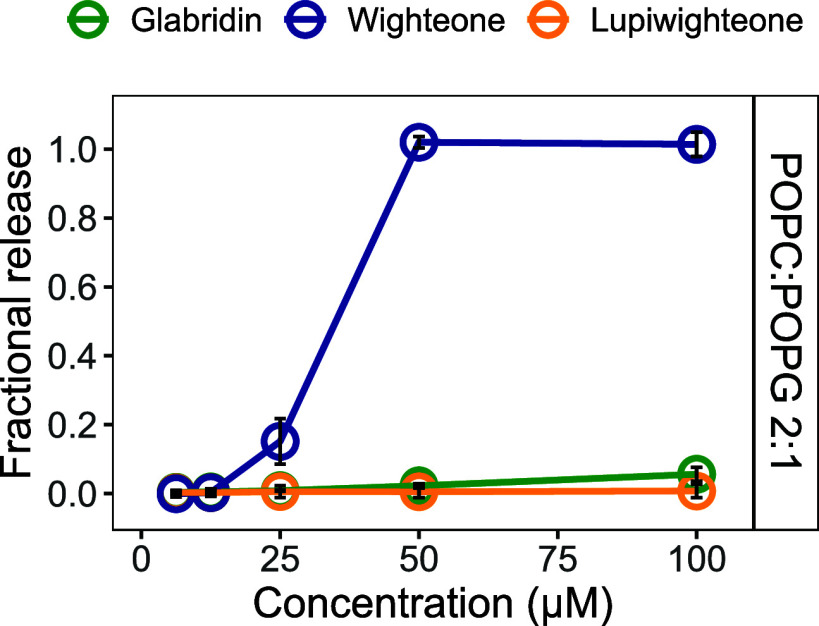
Fractional release of carboxyfluorescein from liposomes formed with POPC:POPG 2:1 after 30 min of incubation with glabridin (green), wighteone (blue), and lupiwighteone (yellow). Data are expressed as averages, and error bars represent the standard deviation of independent replicates (*n* = 3).

Carboxyfluorescein release was also assessed in liposomes with a defined composition of POPC:POPG at a 2:1 molar ratio (Figure 3), a synthetic composition used to simulate a negatively charged membrane.^43^ Notably, the fractional release induced by wighteone with POPC:POPG liposomes was rather comparable to the activity shown with microbial lipid extract liposomes. A concentration of 50 μM wighteone induced complete leakage of carboxyfluorescein from the defined liposomes. However, glabridin did not show permeabilization activity when assessed with POPC:POPG liposomes at concentrations lower than 100 μM. Only limited release (0.1 ± 0.0) occurred in the presence of 100 μM glabridin, representing a striking difference compared to microbial extract liposomes. As shown previously with microbial liposomes, lupiwighteone did not induce any permeabilization in the POPC:POPG liposomes.

### 3.3 MD Simulations of Prenylated Isoflavonoids with Model Membranes

We performed MD simulations to provide information at the atomic level regarding the penetration of the tested prenylated isoflavonoids into the membrane system. Specifically, we calculated the PMF profile from umbrella-sampling MD simulations to estimate the binding affinity for the membrane and the most favorable location of the three prenylated isoflavonoids.

**Figure 4:**
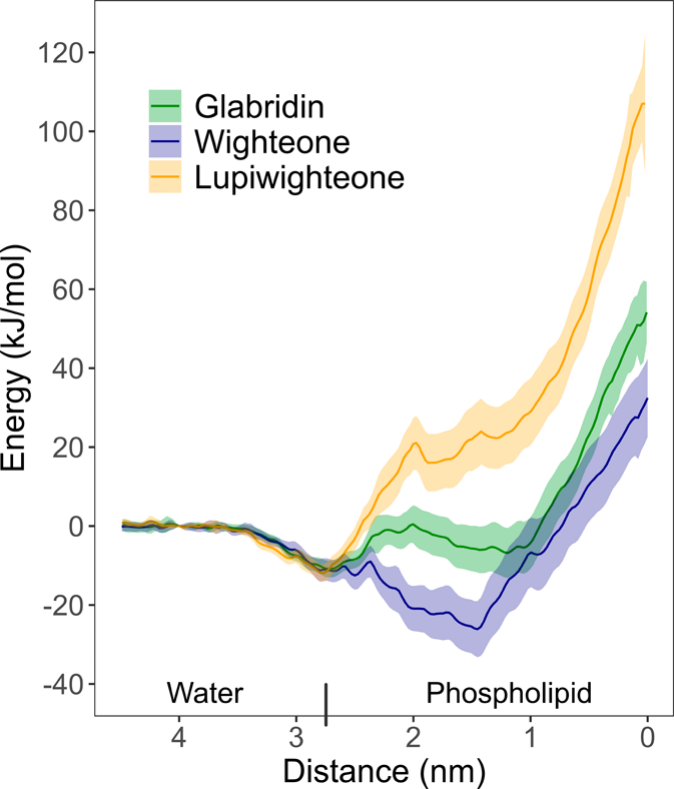
PMF profile of glabridin (green), wighteone (blue), and lupiwighteone (yellow). The distance to the center of mass of the membrane is plotted on the *x*-axis. Lines represent the average energy, and the ribbons represent the deviation standards, calculated using the Weighted Histogram Analysis Method (WHAM) (Hub et al., 2010).^41^

Figure 4 shows the PMF profile of the tested prenylated isoflavonoids from the water region (z = 4.5 nm) to the center of the phospholipid bilayer (z = 0). All the tested prenylated isoflavonoids showed a local free energy minimum toward the surface of the bilayer (z = 2.75 nm), indicating a favorable interaction with the outer part of the phospholipid headgroups. The free energy of wighteone further decreased, reaching a minimum at a 1.45 nm distance from the center of the bilayer. Therefore, the region around 1.45 nm from the membrane can be defined as a preferable location for wighteone. Glabridin showed an energy barrier (increase of energy at around 2.5 nm) to enter the membrane layer and a constant free energy in the region between z = 2 nm and z = 1 nm. Therefore, the PMF profile of glabridin suggests the existence of almost isoergonic conditions between the outer surface and the more hydrophobic interior of the membrane, with an energetic barrier in between. Lupiwighteone showed a clear increase in energy along the phospholipid bilayer. This steep increase in energy delineates a highly unfavorable transfer from the water to the lipid region. Overall, the PMF profiles indicate that partition from the aqueous to the membrane phase is favored for wighteone and unfavored for lupiwighteone, with glabridin having an intermediate behavior. Last, all three compounds are characterized by a (further) increase in free energy in the bulk region of the membrane (around 0 nm). This indicates that the presence of single molecules of these prenylated isoflavonoids in the center of the membrane and their interaction with the phospholipid tails is highly unfavorable, constituting also a kinetic barrier to translocation toward the inner leaflet.

To further investigate the impact of the tested compounds on the membrane structure, we calculated the lipid order parameter and the radial distribution function (RDF) of the phosphate atoms (Figure S7). The RDF data suggest that the presence of prenylated isoflavonoids enhances the ordering of lipid polar heads, which in turn leads to an increase in the order parameters of the aliphatic chains. A similar effect is observed in configurations where isoflavonoids are positioned at 2.75 nm (slightly above the polar heads) and at 1.45 nm (just below), indicating that interactions with the polar heads play a key role in driving the overall reorganization of the bilayer.

### 3.4 Configurations of Prenylated Isoflavonoids When Interacting with Membranes

**Figure 5:**
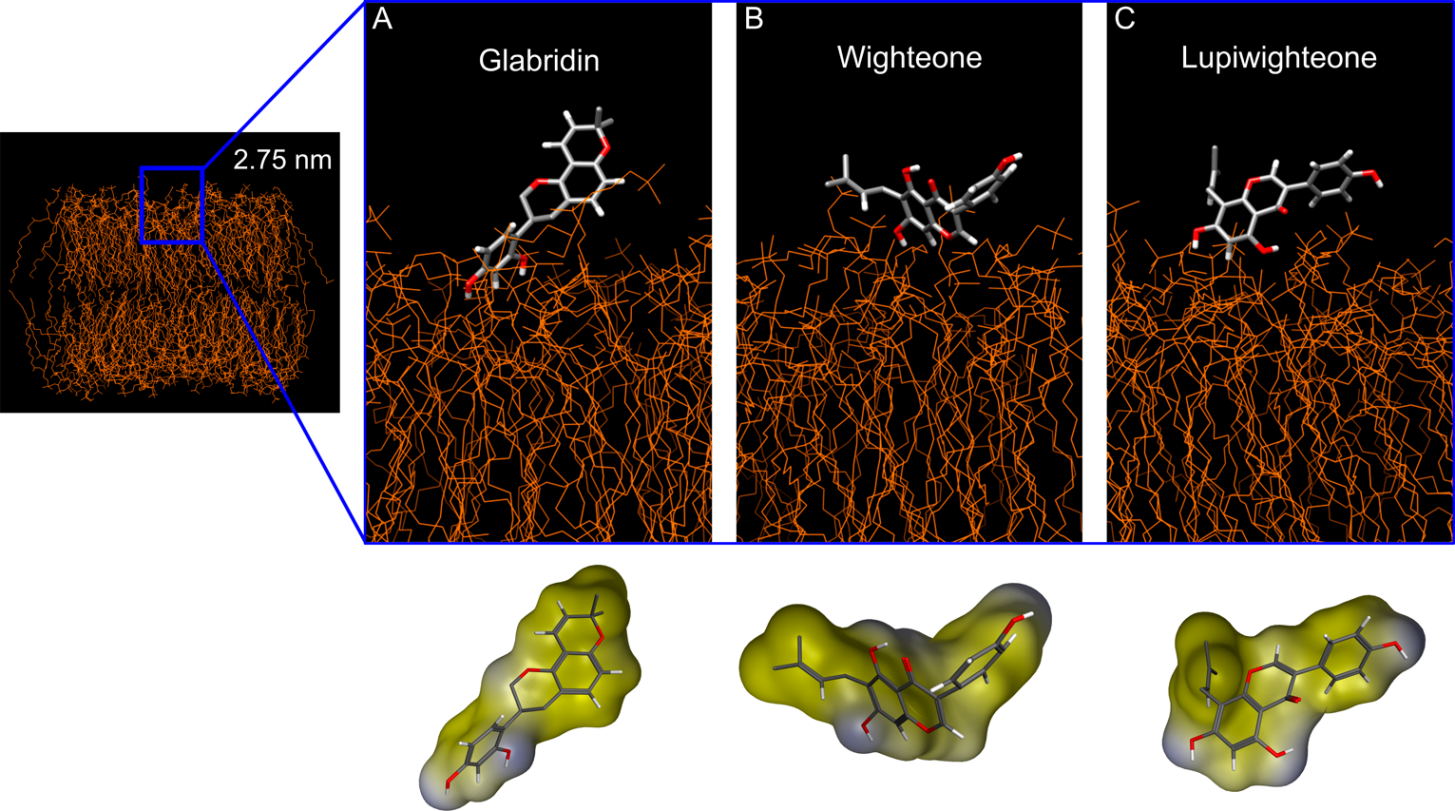
Conformation of glabridin (**A**), wighteone (**B**), and lupiwighteone (**C**) at 2.75 nm distance from the center of mass of the membrane. Most representative conformations of the compound and the membrane as shown in the top part. The colors in the prenylated isoflavonoid structures represent different atoms (gray, red, and white for carbon, oxygen, and hydrogen, respectively). The bottom part shows only the conformation of the prenylated isoflavonoids, highlighting the molecular surface. Yellow indicates the hydrophobic areas, and blue indicates the hydrophilic areas.

Figure 5 shows the most representative conformation of the three tested prenylated isoflavonoids at a distance of 2.75 nm from the center of mass of the membranes (i.e., at the surface of the phospholipid headgroups). The conformations shown resulted from the clustering of frames of the MD simulation where the compounds were at a distance of 2.75 ± 0.05 nm. The top part shows the conformations, and the bottom part shows the hydrophobic and hydrophilic surfaces of the compounds in those conformations, indicating a more defined separation of hydrophilic and hydrophobic regions for lupiwighteone. The atoms that are closer to the membrane surface are the two hydroxyl groups present on the B ring for glabridin (positions *C*2’ and *C*4’), the hydroxyl group present on the A ring for wighteone (position *C*7), and the two hydroxyl groups present on the A ring for lupiwighteone (positions *C*5 and *C*7) (also in accordance with the density profiles shown in Figure S6).

**Figure 6:**
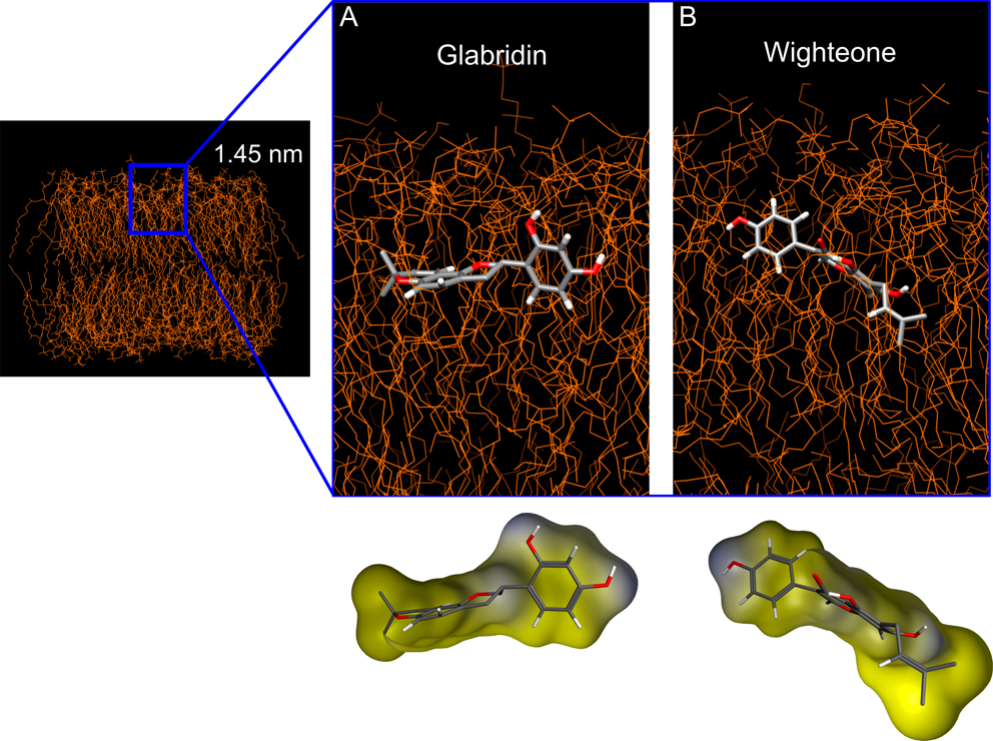
Conformation of glabridin (**A**) and wighteone (**B**) at 1.45 nm distance from the center of mass of the membrane. Most representative conformations of the compound and the membrane, as shown in the top part. The colors in the prenylated isoflavonoid structures represent different atoms (gray, red, and white for carbon, oxygen, and hydrogen, respectively). The bottom part shows only the conformation of the prenylated isoflavonoids, highlighting the molecular surface. Yellow indicates the hydrophobic areas, and blue indicates the hydrophilic areas.

Figure 6 shows the conformations of glabridin and wighteone at a distance of 1.45 nm from the membrane (clustering of frames at 1.45 ± 0.05 nm from the center of mass of the membrane). Lupiwighteone is not shown as, based on the PMF calculations, it is not expected to enter the bilayer (Section 3.3). Glabridin is placed flat, parallel to the bilayer surface. Wighteone is arranged to position the hydroxyl group present on the B ring (position *C*4’) closer to the phospholipid headgroup and the hydrophobic prenyl group in position *C*6 on the A ring deeper in the center of the bilayer (Figure S6).

## 4 Discussion

**Figure 7:**
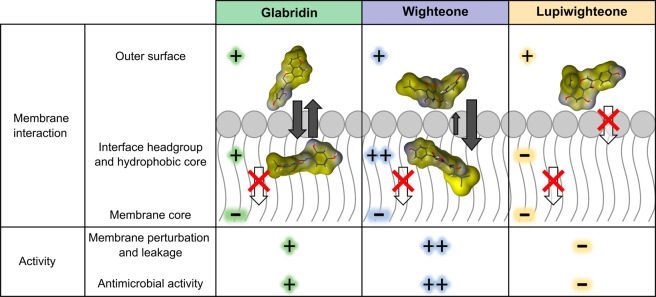
Schematic representation of the main findings of this study. Conformation of glabridin, wighteone, and lupiwighteone on the surface of the membrane, at the interface between the headgroup and the hydrophobic core. Yellow indicates the hydrophobic areas, and blue indicates the hydrophilic areas. The arrows represent the intercalation in the membrane bilayer and the movement toward the core or the membrane; the bigger, the more favorable. Arrows with a red cross indicate nonfavorable movement. + and – are used to describe qualitatively the interactions with the membrane (based on the PMF profiles) and activities (based on the MIC and MBC and leakage assays).

This study aimed to characterize the interaction of selected prenylated isoflavonoids with model (microbial) membranes and relate these results to the observed antimicrobial effects. Therefore, we first used artificial liposomes as model systems to confirm the membrane permeabilization effects previously reported in microorganisms^9,17,44^ and subsequently defined their interactions and position in the membrane by MD simulations. Figure 7 summarizes the main findings of this study in relation to previous observations regarding the antimicrobial activities of glabridin, wighteone, and lupiwighteone.

### 4.1 Glabridin and Wighteone Target Microbial Membranes

Glabridin and wighteone are prenylated isoflavonoids with potent antimicrobial activity. Previous studies reported that glabridin and wighteone induced membrane permeabilization in microbial cells such as *L. monocytogenes*, *E. coli* (when tested with EPI), and in the yeast *Z. parabailii*.^8,9,17^ Moreover, a combination of proteomic and transcriptomic studies and quantitative structure–activity relationship (QSAR) analysis previously proposed that the primary target of these compounds is the lipid bilayer of the membrane.^8,17,45^ In this study, the permeabilization activity was tested on liposomes, indicating that indeed, their activity relies on the interaction with the lipid part and subsequent permeabilization of the microbial membranes. The concentrations of prenylated isoflavonoids that induced the leakage of carboxyfluorescein from liposomes in this study are similar to those tested before, for which permeabilization was shown in microbial cells (59–77 μM). Importantly, the PMF profiles of the three compounds aligned with their permeabilization efficacies, indicating more probable intercalation inside the bilayer for wighteone than for glabridin. The most favorable interaction in the phospholipid bilayer and permeabilization efficacy (liposomes) shown for wighteone could also explain the generally higher antimicrobial activity and permeabilization capacity reported for this compound (Section 3.1).^8,9^

Although in this study no Gram-positive lipid extract was used, *E. coli* liposomes may represent a general bacterial cytoplasmic membrane due to the absence of an outer membrane and also relate to the permeabilization activities shown against *E. coli* in the presence of efflux pump inhibitors. Notably, the model membranes used in this study are devoid of intracellular metabolism and membrane proteins. Therefore, the permeabilization effect can be attributed to the direct interaction of antimicrobial compounds with the lipid bilayer rather than through interactions with other membrane components, such as proteins, or as a secondary effect of another mechanism. The permeabilization of liposomes indicates that the activity of glabridin and wighteone is independent of the energy status of the cell (e.g., membrane potential). Therefore, glabridin and wighteone are expected to be active also against slow-growing or dormant microorganisms, which is an important aspect of their possible applications.

This study indicated that the interaction of glabridin and wighteone inside the phospholipid bilayer occurs at the interface between the headgroup and the hydrophobic core of the bilayer. The movement toward the core of the membranes is highly unfavorable, as indicated by the steep increase in the energy profile around the center of mass of the membrane. A similar preferential location between the headgroup and the hydrophobic core of the bilayer was reported for other phenolic phytochemicals such as quercetin, luteolin, and genistein.^46−51^ This is the first study to report the interaction of glabridin and wighteone with a lipid bilayer. The position of glabridin and wighteone within the bilayer near the headgroup suggests that these interactions are pivotal for their membrane perturbation effects, as previously proposed for other flavonoids.^48,49^

### 4.2 The Activity of Glabridin May Require a More Complex Membrane Architecture

Glabridin showed a clear difference in permeabilization efficacy between lipid extract (*E. coli* and *S. cerevisiae*) liposomes and POPC:POPG liposomes. The lower permeabilization efficacy shown for POPC:POPG liposomes may result from different membrane properties, e.g., different surface charge of the membrane, spatial configuration of the phospholipids inside the membrane (e.g., formation of lipid domains)^52^ and different shape of the phospholipids in the mixture. Liposomes with a POPC:POPG 2:1 composition were used to simulate a negatively charged membrane as the one present in bacterial membranes and in liposomes made with *E. coli* extract (Table S1, 15.1% PG and 9.8% of cardiolipin). Therefore, we do not foresee that differences in surface charge between liposomes made with E. coli and POPC:POPG can fully explain the difference in the permeabilization efficacy of glabridin. Previous studies have shown how phospholipid composition, formation of specific lipid domains, and shape of phospholipid can affect the membrane interaction of antimicrobial agents, such as antimicrobial peptides.^53−57^ Interestingly, a previous study highlighted the impact of sterols and sphingolipid biosynthesis on the susceptibility of *S. cerevisiae* to glabridin.^45^ Sterols and sphingolipids are known to induce lipid microdomains in the membrane^58−60^ and are potentially relevant for the interaction of glabridin with yeast membranes. Moreover, the formation of lipid microdomains has also been reported in bacterial membranes in the absence of sterols.^61^ Therefore, lipid microdomains may influence the activity of glabridin and may explain the lower permeability in a more simplified system such as the POPC:POPG liposomes. Further studies are needed to gain a better understanding of the activity of glabridin in relation to membrane composition and architecture.

### 4.3 The Position of the Prenyl Group Defines the Membrane Interaction Properties

This study further investigated the impact of the position of the prenyl group on the antimicrobial efficacy of prenylated isoflavonoids by comparing wighteone and lupiwighteone. Previous studies have shown that lupiwighteone is not active using whole-cell assays;^8−10^ however, the cause of this inactivity remained unclear. The absence of permeabilization of liposomes upon exposure to lupiwighteone, in addition to the low penetration capacity into membranes measured by the PMF, can help explain the lack of antimicrobial activity of this prenylated compound. Although not all of the mechanistic details have been clarified, the results of this study clearly indicate that the change in the prenyl position directly affects the interactions with the membrane and the permeabilization effects.

The antimicrobial inactivity of lupiwighteone was previously tested only on metabolically active microbial cells. Importantly, this study can rule out the possibility that the inactivity is due to resistance mechanisms or metabolic processes from microbial cells, such as active removal or export from the microbial cells. This indication is also supported by the fact that an efflux pump inhibitor did not improve the antimicrobial activity of this compound.^8^ Therefore, it is proposed that the lack of activity of lupiwighteone is due to the unfavorable intercalation toward the center of the lipid bilayer. Importantly, this study helps to explore the key chemical features of prenylated isoflavonoids for their antimicrobial activity, which can also support explorative research to discover novel antimicrobial compounds.

### 4.4 Surface Properties of Prenylated Isoflavonoids in Relation to Antimicrobial Activities

The calculation of the PMF profile of glabridin, wighteone, and lupiwighteone indicated that the interaction between the compounds and the surface of the membrane (headgroup) is favorable for all three compounds. Conformations of the prenylated compounds on the surface of the membranes showed that the more hydrophilic parts of the molecules interact with the polar head groups. The hydrophilic area seems to drive the interaction of the compounds with the surface of the membranes outside of the actual bilayer. An important feature of prenylated isoflavonoids for their activity is the occurrence of hydrophilic and hydrophobic surface area regions in the molecule, as previously indicated in QSAR studies. The importance of the distribution of hydrophobic area was previously indicated by the QSAR study of Araya-Cloutier et al. (2018),^8^ in which the amphiphilic moment (in terms of vsurf_A descriptor) was negatively correlated with the antimicrobial activity of prenylated (iso)flavonoids. The values of the molecular descriptor vsurf_A indeed follow an opposite trend compared to the leakage shown in liposomes (vsurf_A values: wighteone 2.26, glabridin 3.73, and lupiwighteone 4.17). Interestingly, hydrophobicity cannot alone explain the difference in activity. The position of the prenyl group does not influence the predicted partition coefficient (LogP_(o/w)_ values: 4.2 for glabridin and 2.9 for wighteone and lupiwighteone). Combining this information with the leakage experiment and the PMF profile (at 1.45 nm distance) indicates that the defined separation of hydrophilic and hydrophobic regions can negatively impact the intercalation along the membranes and the permeabilization activity of prenylated isoflavonoids. The higher amphiphilicity shown for lupiwighteone may result in lower antimicrobial activity due to different configurations of this compound at the membrane surface and lower overall partitioning into the bilayer. On the other hand, the lower amphiphilicity reported for glabridin and wighteone may allow better intercalation of these compounds in the bilayer.

Importantly, our study used MD simulations to explore the behavior of these prenylated isoflavonoids as single molecules and not as aggregates. As the formation of aggregates in solution and in membranes was previously reported for other prenylated phenolics,^29^ further MD simulations and characterization of aggregate formation of prenylated isoflavonoids in solution and interaction with membranes will provide further information on the permeabilization efficiency of these compounds.

In conclusion, this study assessed the permeabilization activity and interactions with membranes of glabridin, wighteone, and lupiwighteone using *in vitro* and *in silico* model membranes. The permeabilization effects shown in liposomes confirmed the lipid component of the membrane as the primary target for glabridin and wighteone as antimicrobial compounds. MD simulations complemented the information from the permeabilization assays, indicating a more favorable intercalation toward the center of the membrane for wighteone than glabridin. Moreover, the inability of lupiwighteone to induce permeabilization in model membranes and the unfavorable location inside the bilayer might provide an explanation for its inactivity as an antimicrobial compound. Overall, this study contributed to the elucidation of the mode of action of these antimicrobial compounds, which is a key aspect in unlocking their possible applications.

## Supporting Information Available

Phospholipid profile of *E. coli* and *S. cerevisiae* lipid total extract; details of the system used for MD simulations; DLS measurements of liposomes; fractional release of carboxyfluorescein from liposomes, including negative controls; and density profiles of prenyl and hydroxyl groups on selected conformations (PDF)
