# Supplementary material for: Mechanistic Insights into the Membrane Permeabilization Activity of Antimicrobial Prenylated Isoflavonoids: A Comparative Study of Glabridin, Wighteone, and Lupiwighteone
Source: J Agric Food Chem. 2025 Mar 5;73(11):6668–77. doi: 10.1021/acs.jafc.5c01688 (PMC11926876; doi:10.1021/acs.jafc.5c01688)
Supplement: Supplementary file 1 — jf5c01688_si_001.pdf [file jf5c01688_si_001.pdf]

Supporting Information

Mechanistic insights into the membrane permeabilization activity of antimicrobial prenylated isoflavonoids: a comparative study of glabridin, wighteone and lupiwighteone

Alberto Bombelli[1,2], Paolo Calligari[3], Gianfranco Bocchinfuso[3], Jean-Paul Vincken[2], Tjakko Abee[1], Heidy M.W. den Besten[1], Lorenzo Stella[3] and Carla Araya-Cloutier[2*]

[1]Food Microbiology, Wageningen University & Research, 6700 AA Wageningen, the Netherlands

[2]Food Chemistry, Wageningen University & Research, 6700 AA Wageningen, the Netherlands

[3]Department of Chemical Science and Technologies, Tor Vergata University of Rome, 00133 Rome, Italy

***Email:** carla.arayacloutier@wur.nl

Table S1. Phospholipid profile of *E. coli* and *S. cerevisiae* lipid total extract, provided by Avanti Polar Lipids (Alabaster, Al, USA)

Extract	Component	Percentage (w/w)
E. coli	Phosphatidylethanolamine	57.5
	Phosphatidylglycerol (PG)	15.1
	Cardiolipin	9.8
	Unknown	17.6
S. cerevisiae	Phosphatidylcholine (PC)	18.7
	Phosphatidylinositol	13.3
	Phosphatidylethanolamine	4.5
	Phosphatidylserine	4.4
	Lysophosphatidylethanolamine	1.9
	Phosphatidic acid	1.1
	Lysophosphatidylcholine	0.6
	Phosphatidylglycerol	0.5
	L-A-Glycerophosphorylcholine	0.1
	Unknown	54.6

.

Table S2. Details of the systems used for the MD simulations based on the prenylated isoflavonoids tested

**Compound**	Lipid molecules (No.)	Water molecules (No.)	Sodium ions. (No.)	Box dimensions (nm)
**Glabridin**	128	9110	42	5.9 x 5.9 x 12.5
**Wighteone**	128	9111	42	5.9 x 5.9 x 12.5
**Lupiwighteone**	128	9105	42	5.9 x 5.9 x 12.5

Table S3: Details of the temperature profile used during the MD simulations of each window during the umbrella sampling. The simulated annealing step was used to equilibrate the system and the production run step was used to calculate the PMF profile using the Weighted Histogram Analysis Method (WHAM)

Step	Time range (ns)	Temperature (°C)
Simulated Annealing	0-2	25
	2-2.1	101.85
	2.1-3	26.85
	3-5	26.85
	5-5.1	36.85
	5.1-6	26.85
	6-8	26.85
	8-8.1	36.85
	8.1-9	26.85
	9-11	26.85
	11-11.1	36.85
	11.1-12	26.85
	12-14	26.85
	14-14.1	36.85
	14.1-15	26.85
	15-17	26.85
	17-17.1	36.85
	17.1-18	26.85
	18-20	26.85
	20-25	25
Production run	25-40	25

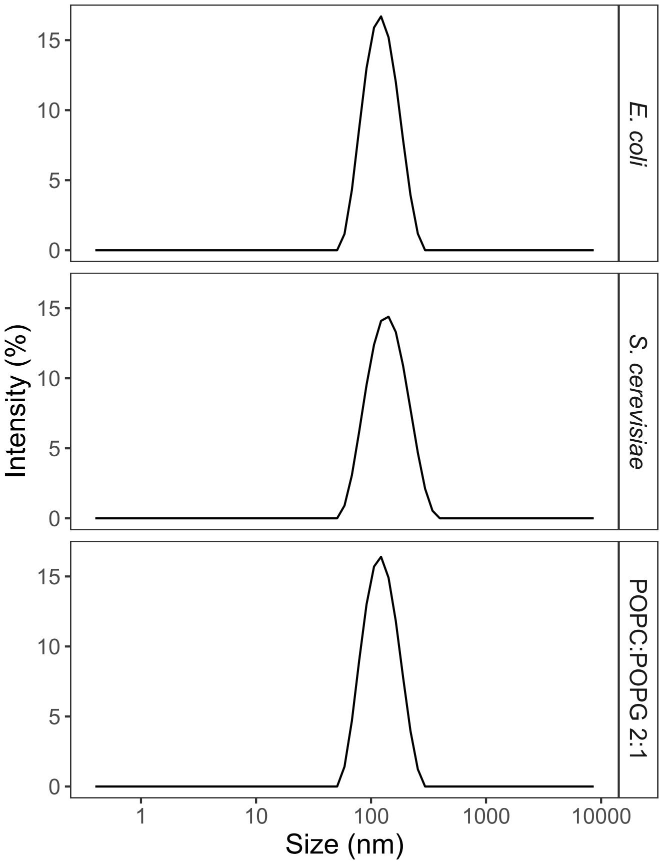

Figure S1. Size of liposomes after extrusion through 100 nm polycarbonate membrane measured by DLS. Representative measurements are shown for liposomes prepared with *E.coli* extract (**top**), *S. cerevisiae* extract (**center**) and POPC:POPG 2:1 (**bottom**).

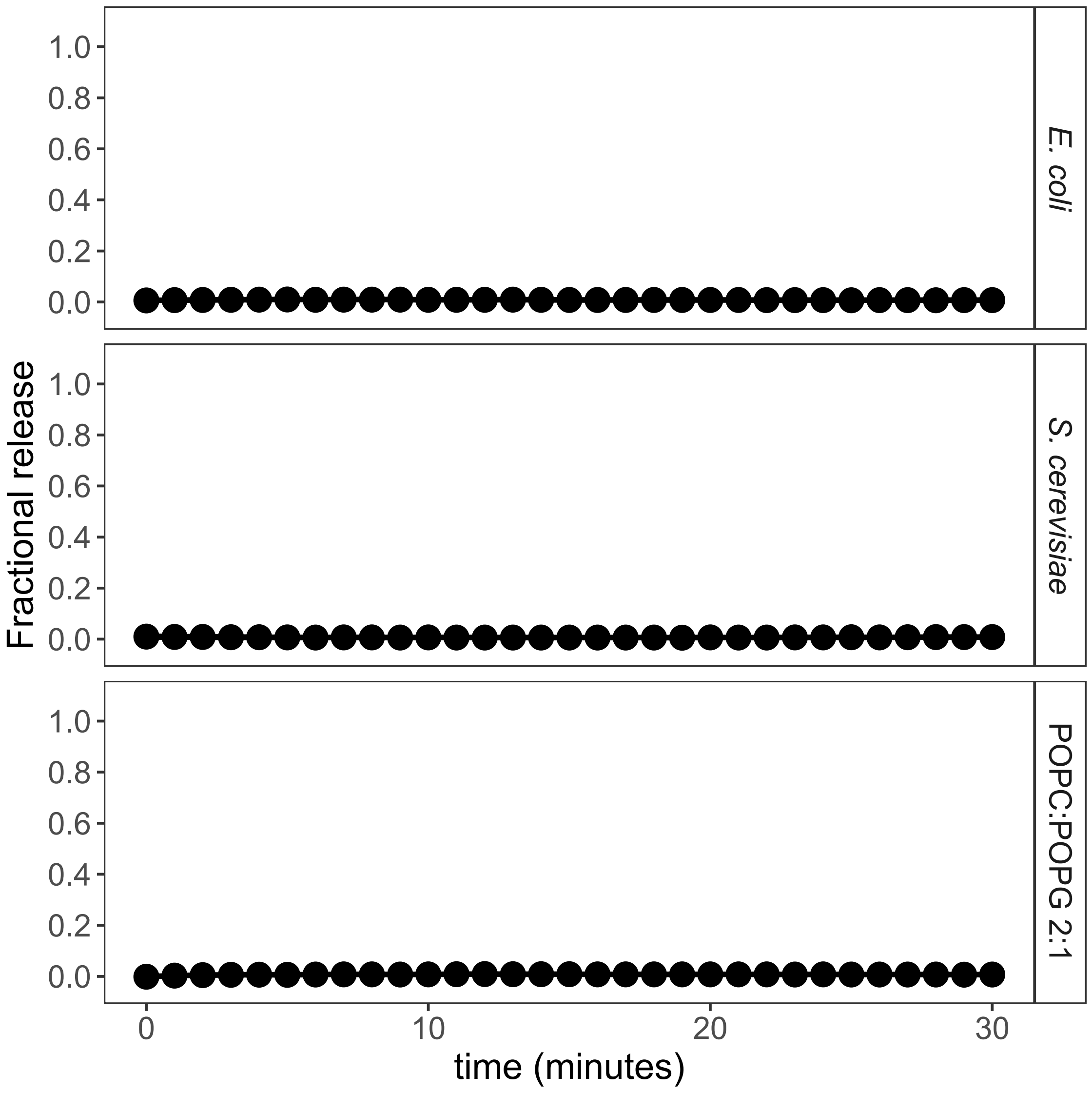

Figure S2. Fractional release of carboxyfluorescein from liposomes prepared with *E.coli* extract (**top**), *S. cerevisiae* extract (**center**) and POPC:POPG 2:1 (**bottom**) during the incubation with 1% of DMSO. Data are expressed as averages of independent replicates.

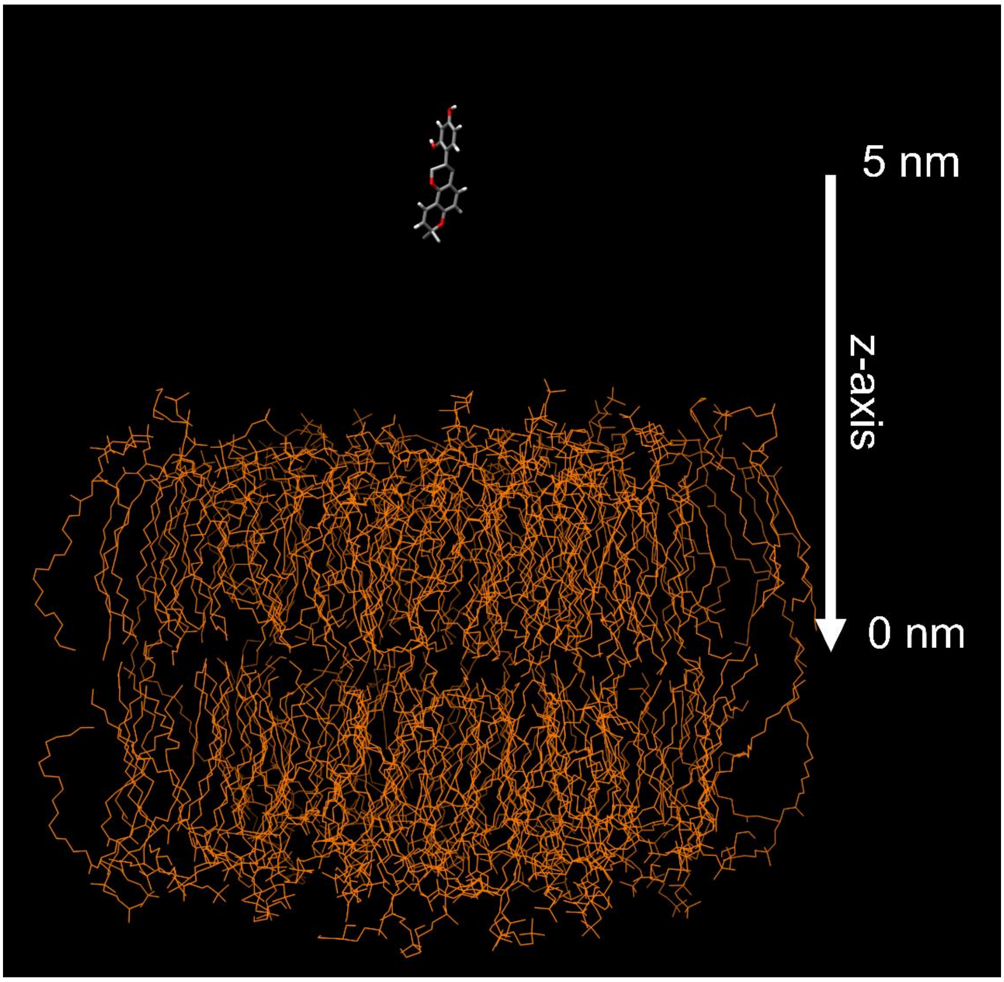

Figure S3. Representation of the system used for the calculation of the PMF. Prenylated isoflavonoids were placed at 5 nm distance from the centre of mass of the membrane (based on z-axis). The potential of mean force (PMF) profiles were calculated from 102 windows which were generated from the trajectory from z = 4.55 to z = -0.5 nm. Glabridin was used as an example in this figure.

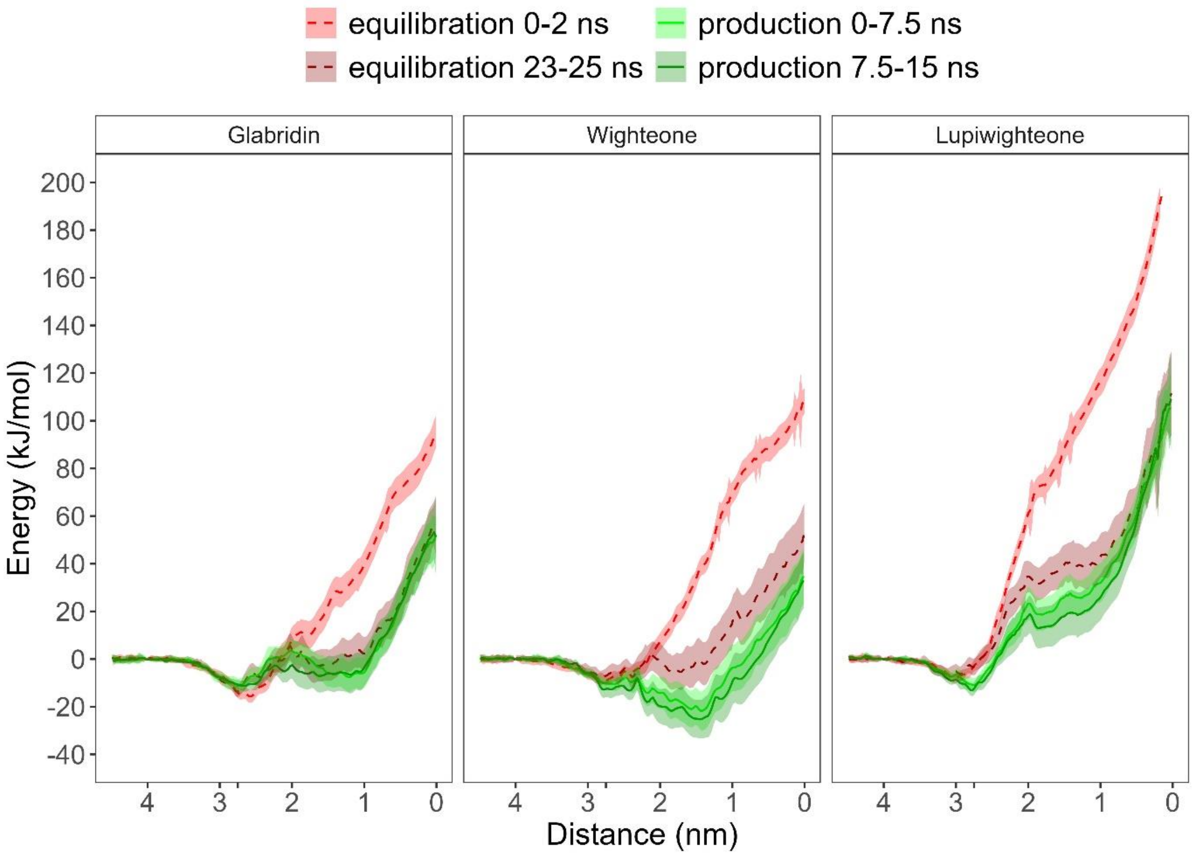

Figure S4. PMF profile of glabridin (left), wighteone (center) and lupiwighteone (right). The PMF was calculated before the annealing (equilibration 0-2 ns), after the annealing (equilibration 23-25 ns) and during the production step in two time frames (production 0-7.5 ns and production 7.5-15 ns). The distance to the centre of mass of the membrane is plotted on the x-axis. Lines represent the average energy, and the ribbons represent the deviation standards, calculated using the Weighted Histogram Analysis Method (WHAM).

The production run was divided in two ranges to show the stability of the system during the 15 ns of production as indicated by the overlap between PMF profile calculated using the first 7.5 ns of production and the last 7.5 ns of production (production 0-7.5 ns and production 7.5-15 ns, respectively). Moreover, this figure shows the effect of the equilibration step on the system. The convergence of the PMF profile of the last 2 ns of equilibration (equilibration 23-25 ns) to the PMF profiles calculated in the production phase indicates that the system was equilibrated during the production and therefore suitable to calculate the PMF as shown in Figure 4 in the main text of the manuscript.

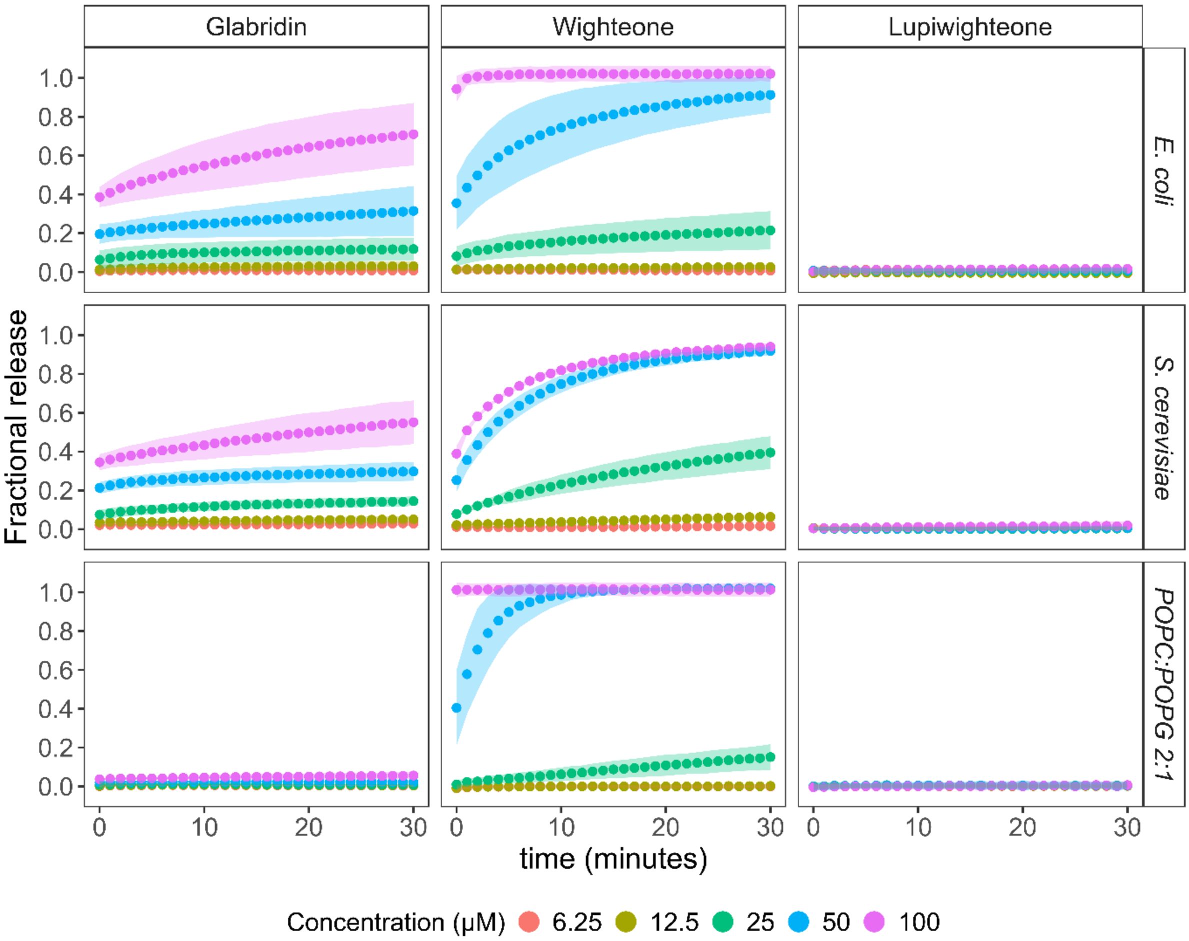

Figure S5. Fractional release of carboxyfluorescein from from *E.coli* (**top**), *S. cerevisiae* (**center**) and POPC:POPG 2:1 (**bottom**) liposomes during the incubation with glabridin (**left**), wighteone (**centre**) and lupiwighteone (**right**). Different concentrations of prenylated isoflavonoids (6.25 – 100 μM) are represented with different colours. Data are expressed as averages and shades represent the standard deviation of independent replicates ($n = 3$).

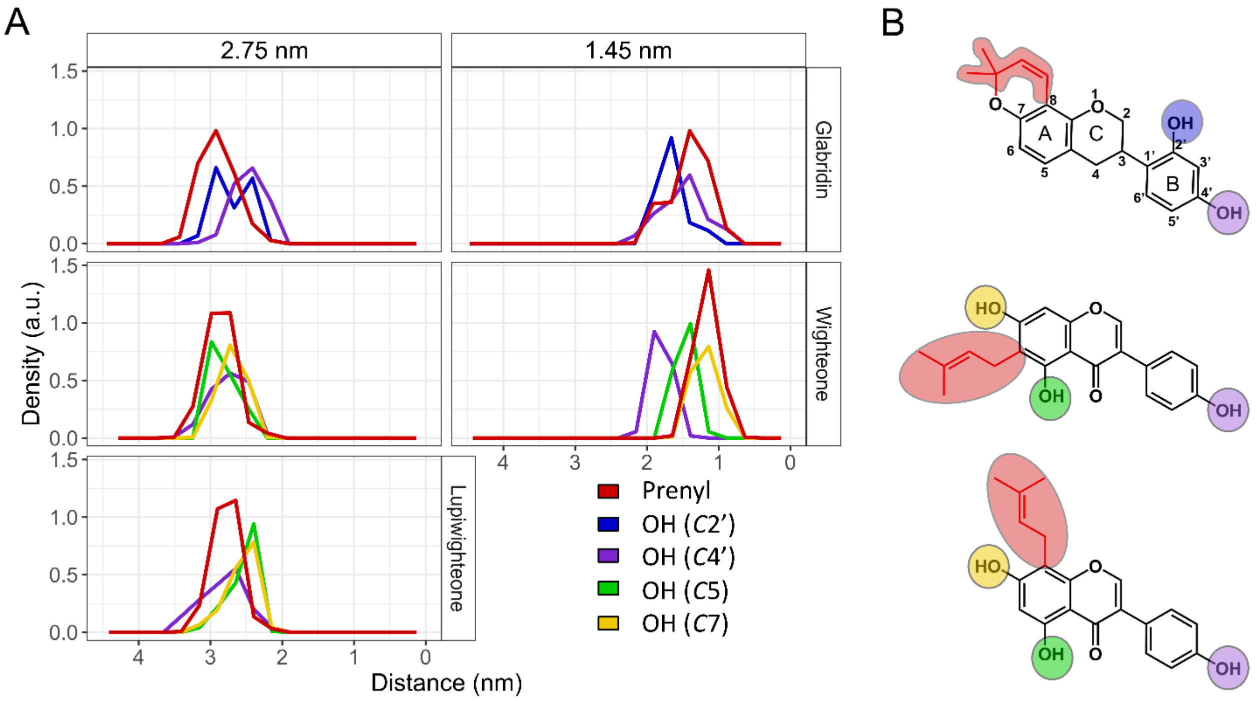

Figure S6. A) Density profiles of prenyl and hydroxyl groups of glabridin, wighteone and lupiwighteone in the frames selected for the clustering analysis. The conformations were selected based on the distance from the membrane; specifically, frames where the center of mass of the prenylated isoflavonoids were at a distance of 2.75 ± 0.05 nm (left) and 1.45 ± 0.05 nm (right) were retrieved. The density profile was calculated with Gromacs and divided based on the number of atoms present in the groups. The x axis represents distance from the c.o.m. of the membrane. **B)** The chemical structure of the prenylated isoflavonoids and the subclass-dependent IUPAC numbering is shown on the right. In the chemical structures, the groups used for the density profile are highlighted with corresponding colours used in the density profile graphs.

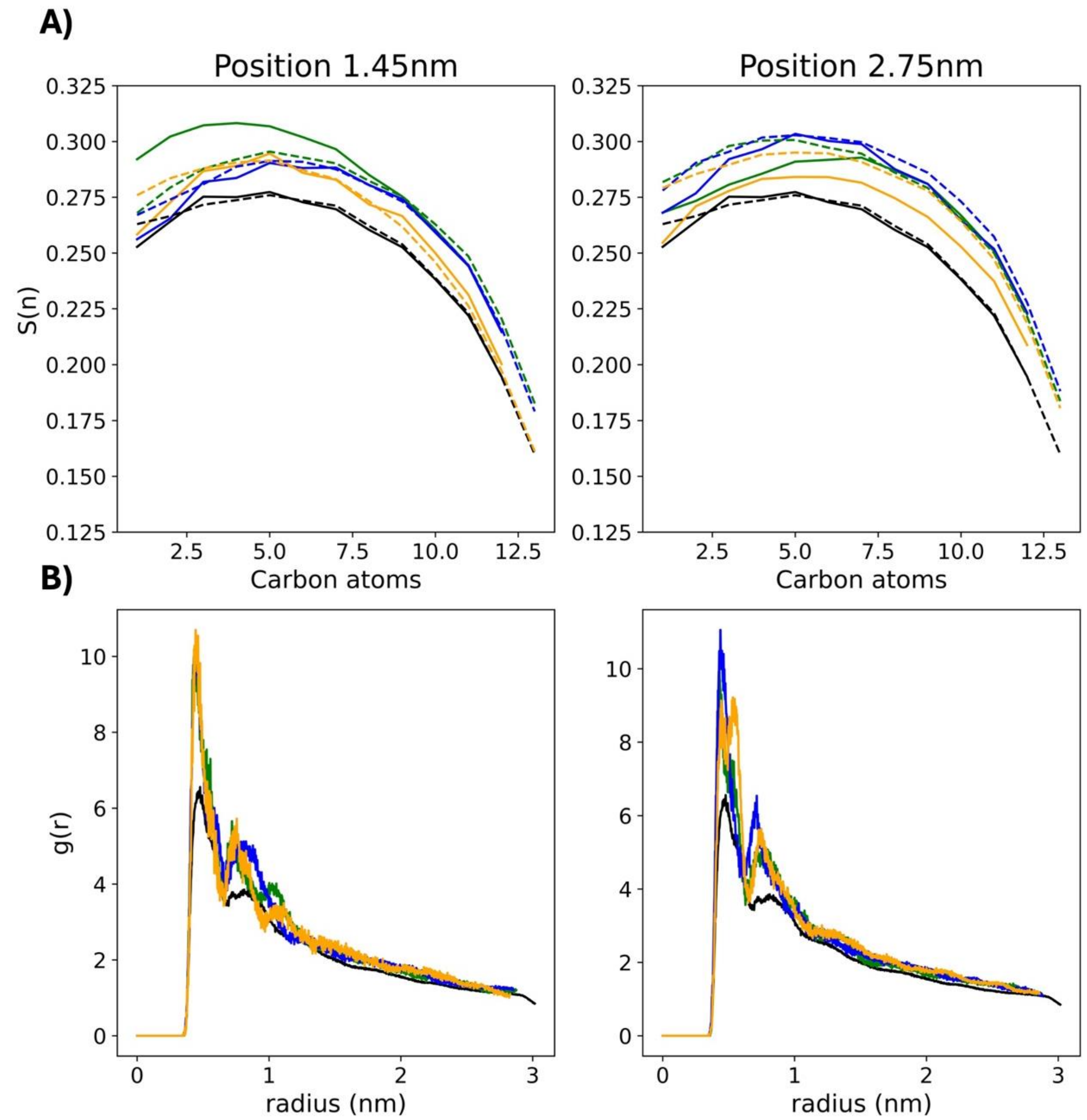

Figure S7: Effects of glabridin, wighteone, and lupiwighteone on membrane structure. **Panel A)** Order parameter (OP), *S(n)*, for the sn1 chain of POPG (solid lines) and POPC (dashed lines) for glabridin (green), wighteone (blue), and lupiwighteone (yellow) at distances of 1.45 nm (left) and 2.75 nm (right) from the membrane center. Here, $S(n) = \frac{1}{2}\langle 3cos^2\theta_n - 1\rangle$, where θ_n is the angle between the n-th C-H bond vector and the bilayer normal. while $\langle ... \rangle$ represent temporal averages along the trajectory and on all lipids of interest. In all cases, the presence of the compounds increases OP compared to the membrane-only simulation (black lines), indicating reduced phospholipid chain mobility. Among the three compounds, lupiwighteone appears to have a weaker effect on OP than glabridin and wighteone. The observed increase in membrane order may result from reduced lipid mobility due to the presence of prenylated isoflavonoids, affecting regions both above (2.75 nm) and below (1.45 nm) the lipid polar heads. To further investigate this, **Panel B)** presents the radial distribution function (RDF), *g(r)*, of phosphorus atoms, which supports this trend. A higher degree of structural order is evident in the presence of the compounds. Here, *g(r)* is the ratio of the average local number density of phosphorus atoms at a distance *r*, to their bulk density. Both OP and RDF were calculated for lipids with a center of mass within 2 nm of the isoflavonoid molecules.
